# Supplementary material for: CD4+ cell count recovery after initiation of antiretroviral therapy in HIV-infected Ethiopian adults
Source: PLoS One. 2022 Mar 24;17(3):e0265740. doi: 10.1371/journal.pone.0265740 (PMC8947242; doi:10.1371/journal.pone.0265740)
Supplement: S1 Table — (DOCX) [file pone.0265740.s002.docx]

**S1 Table: Baseline characteristics of patients included and excluded from the analysis**

| **Characteristics** | **Included**  **(N = 566)** | **Excluded**  **(N = 194)** | ***P–value*** |
| --- | --- | --- | --- |
| Age (year), median (IQR) | 36 (29–42) | 35 (28–40) | 0.350 |
| Sex, n (%) |  |  | 0.555 |
| Male | 242 (42.8) | 89 (45.9) |  |
| Female | 324 (57.2) | 105 (54.1) |  |
| WHO clinical stage, n (%) |  |  | 0.311 |
| 1/2 | 439 (77.6) | 145 (74.7) |  |
| 3/4 | 127 (22.4) | 49 (25.3) |  |
| CD4+ cell count (Cells/mm^3^), median (IQR) | 264 (192–500) | 261 (188–562) | 0.248 |
| Tuberculosis, n (%) | 37 (6.5) | 15 (7.7) | 0.569 |
| Hemoglobin (g/dl), median (IQR) | 12.6 (11.4–14.7) | 13.5 (12.0–14.8) | 0.130 |
